# Corporate health culture promotes infection control measures against COVID‐19 in the workplace

**DOI:** 10.1002/1348-9585.12373

**Published:** 2022-11-24

**Authors:** Tomohisa Nagata, Kiminori Odagami, Masako Nagata, Koji Mori

**Affiliations:** ^1^ Department of Occupational Health Practice and Management Institute of Industrial Ecological Sciences, University of Occupational and Environmental Health, Japan Kitakyushu Japan; ^2^ Department of Occupational Medicine School of Medicine, University of Occupational and Environmental Health Kitakyushu Japan

**Keywords:** corporate culture, COVID‐19, health and productivity management, health culture, infection, workplace

## Abstract

**Objectives:**

The present study examined the relationship between health culture evaluated by the Health and Productivity Survey Sheets and the implementation status of infection control measures against COVID‐19 in the workplace.

**Methods:**

This was a cross‐sectional study using the corporate data (2518 companies) collected for the purpose of selecting the excellent company of health and productivity management by the Japanese government. The explanatory variable was the overall evaluation score, and the outcome was whether or not infection control measures against COVID‐19 in the workplace. We used logistic regression analysis and calculated the odds ratio adjusted for the industry sector, the corporation size, and the operating profit ratio by the overall evaluation score category.

**Results:**

The odds ratio of all infection control measurements in the workplace increased as the evaluation score increased.

**Conclusions:**

This study revealed a new finding that the presence of a healthy culture in the workplace will lead to the appropriate implementation of infection control measures during a pandemic. The company's ordinary commitment to employee health will be effective even in times of health crisis, such as during the outbreak of the pandemic.

## 1 INTRODUCTION

The coronavirus disease 2019 (COVID‐19), an infection caused by SARS‐CoV‐2, has spread rapidly worldwide since it was first reported in December 2019 and has continued through several waves in different countries.

The infection routes of the virus are contact, droplet, and aerosol, and workplaces where many people come into contact frequently are one of the places where the risk of infection is high, and it has been suggested that combined infection control measures that take these pathways into account are effective in preventing infection in the workplace.^1^ Furthermore, it has been suggested that infection control in the workplace is not only directly effective but also influences the infection behavior of the workers,^2^, ^3^ makes them feel protected by the organization,^4^ and maintain good mental health condition.^5^, ^6^

There are significant differences among workplaces in the implementation of infection control measures. It was reported that the larger the size of the company, the more measures are implemented in Italy, Japan, and Iran.^7^, ^8^, ^9^ It was also reported that the financial sector took more progress measures than the food sector in Italy.^7^ However, to the best of our knowledge, the impact of factors related to workplace health cultures, such as workplace health promotion policies and practices, has not been examined.

Health culture is defined as “the creation of a working environment where employee health and safety is valued, supported and promoted through workplace health programs, policies, benefits, and environmental supports. Building a culture of health involves all levels of the organization and establishes the workplace health program as a routine part of business operations aligned with overall business goals.”^10^ Several proposals have been made for the components of health culture, and many attempts have been made to evaluate them.^11^, ^12^, ^13^, ^14^, ^15^ However, because the components are multifaceted and complex concepts, each researcher has created them in an exploratory manner, and there is currently no unified evaluation scale or evaluation method. Also, because of differences in racial and national cultural backgrounds, it is considered necessary to exercise caution in introducing foreign indicators and tools directly to one's own country or organization. The relationship between the existence of health culture and health‐related outcomes of employees, such as employee awareness of and participation in the health promotion programs provided by the organization,^16^, ^17^ health‐related behavioral changes,^18^ self‐evaluated performance, altruism, and happiness through health behaviors,^19^ employee job performance and satisfaction, and turnover has been examined.^13^

The Health and Productivity Management (HPM) Survey conducted in Japan can also be positioned as a tool for measuring corporate health culture. The Japanese government is implementing policies to extend the working lives of the elder population in response to the decline in the working‐age population,^20^ and extending healthy life expectancy has become an important issue in this context. The government has been developing initiatives of HPM, which encourages corporations to invest in the health of their employees.^21^ One of the main programs in the initiatives is a recognition program for corporations promoting HPM. And what is used for this evaluation is the HPM Survey Sheets. In the HPM Survey, the participating corporations are evaluated in four areas: “positioning of OHM in the corporation's philosophy and policies,” “organized frameworks,” “specific systems for implementing HPM,” and “assessment and improvement.” The deviation value of each corporation is calculated for each area, and the corporations are ranked by their overall evaluation. In the 2020 HPM Survey, survey items on the implementation status of infection control measures against COVID‐19 measures were added.^22^

The purpose of this study is to examine the relationship between corporate health culture measured with the HPM survey and the implementation status of infection control measures by the company unit in Japan.

## 2 METHODS

This was a cross‐sectional study. In the HPM recognition programs, participating corporations voluntarily downloaded the HPM Survey Sheets from the Ministry of Economy, Trade, and Industry's website, filled in the answers, and submitted them.

Corporations in the HPM Stock Selection and the Certified HPM Corporation Recognition Program in the large corporation sector are evaluated using self‐administered HPM Survey Sheets. We obtained the results (company's number = 2523) from each corporation's response to the 2020 HPM Survey Sheets. Any corporation above a certain size in Japan can apply for this survey. For example, for the companies representing 92% of the applicants (2311 companies), the definition of the certain size is that the company has at least 51 employees in the retail industry, at least 101 employees in the wholesale and service industry, and at least 301 employees in the manufacturing and other industries. Since there are approximately 11 000 corporations in 2016 Japan that meet this definition,^23^ this means that 23% of the corporations have applied.

In this study, we analyzed the completed data (*n* = 2359) excluding the data of the 164 companies that contain missing values. The details of the composition of the questionnaire have been previously published.^11^

### 2.1 Explanatory variables

The HPM Survey Sheets evaluate four areas: positioning of HPM in the corporation's philosophy and policies; organized frameworks; specific systems for implementing HPM; and assessment and improvement. Each company has a deviation value calculated for each of these four evaluation axes. The overall evaluation score is also calculated using the deviation of four areas. We used the overall evaluation score which is a weighted average of “positioning of HPM in the corporation's philosophy and policies,” “organized frameworks,” “specific systems for implementing HPM,” and “assessment and improvement,” with 3:2:2:3 in the recognition programs. We divided the overall evaluation score into three equal groups and created low‐, moderate‐, and high‐score categories.

### 2.2 Outcome variables

The outcome variables were whether or not preventive measures against COVID‐19 were implemented in the company. The HPM Survey Sheets include some questions that are not used to calculate the overall evaluation score. The items of preventive measures against COVID‐19 in the workplace were questions that are not used in the evaluation, and that was clearly described in the survey sheets so that the applicant companies could know this.

Infection control measures were divided into three categories of questions. The first category, measures to reduce the risk of infection among employees who are forced to come to work, was asked to select all of the following measures that are being implemented: “apply a different shift system than normal to reduce opportunities for contact among employees;” “encourage off‐peak commuting through staggered work hours and flextime to reduce the risk of infection during commuting;” “distribution of masks required for work and commuting;” “checking and reporting health status (temperature check, etc.);” “special considerations for employees with underlying medical conditions that pose a high risk of serious illness in the event of infection.” The second category, engineering control in the workplace environment, was asked to select all of the following measures that are being implemented: “spacing of seats, partitions, and other spatial devices;” “disinfecting areas that many people touch, such as handrails and free‐address desks;” “check and improve ventilation conditions in offices and break rooms.” The third category, other measures, was asked to select all of the following measures that are being implemented: “granting of special leave for employees with children in school due to school closures;” “provision of information on preventive measures against infectious diseases.” For each infection control measure, we used a binary variable of “yes/no.”

### 2.3 Other variables

The infection control measurements were expected to be influenced by the industry and size of the corporation. We, therefore, used information from the questionnaire about industrial classification and the number of full‐time employees as adjustment variables. The HPM Survey Sheets asked the type of industry based on the classification of the Securities Identification Code Committee in Japan, and we recorded them into the 12 classifications based on the International Standard Industrial Classification^24^: agriculture, forestry, fishing, and mining; construction; manufacturing; electricity, gas, heat supply, and water; transport and postal activities; information and communications; wholesale and retail trade; finance and insurance; real estate and goods rental and leasing; medical, health care, and welfare; services; others. The number of full‐time employees was classified as the following five categories: −99; 100–299; 300–999; 1000–2999; and 10 000‐.

A company's health strategy may also be affected by its financial situation. Therefore, as a financial indicator, we used the operating profit ratio, that is the operating profit divided by net sales, and then multiplied by 100.

### 2.4 Statistical analysis

We drew a histogram and calculated the mean, median, and standard deviation of the overall evaluation score. In order to compare the status of implementation of basic occupational health services, we compared the implementation rates of annual health check‐ups and stress‐check, which are required by law in Japan, in three categories of the overall evaluation score.

We used logistic regression analysis to examine the relationships between each evaluation‐score category and each infection control measurement. We calculated the odds ratio and 95% confidential interval of moderate‐ and high‐score categories adjusted for the industry sector, the corporation size, and the operating profit ratio referring to the low‐score category. We used Stata release 16.1 (StataCorp LLC) for all analyses.

### 2.5 Ethical considerations

The Ministry of Economy, Trade, and Industry obtained consent from all responding companies to use these data for research purposes. We also signed a written commitment with the Ministry of Economy, Trade, and Industry to ensure that the data would be kept within the institution and to confirm that we would not disclose the results in a form that made it possible to identify any individual corporation. Additionally, we did not handle individuals' personal information.

## 3 RESULTS

**FIGURE 1:**
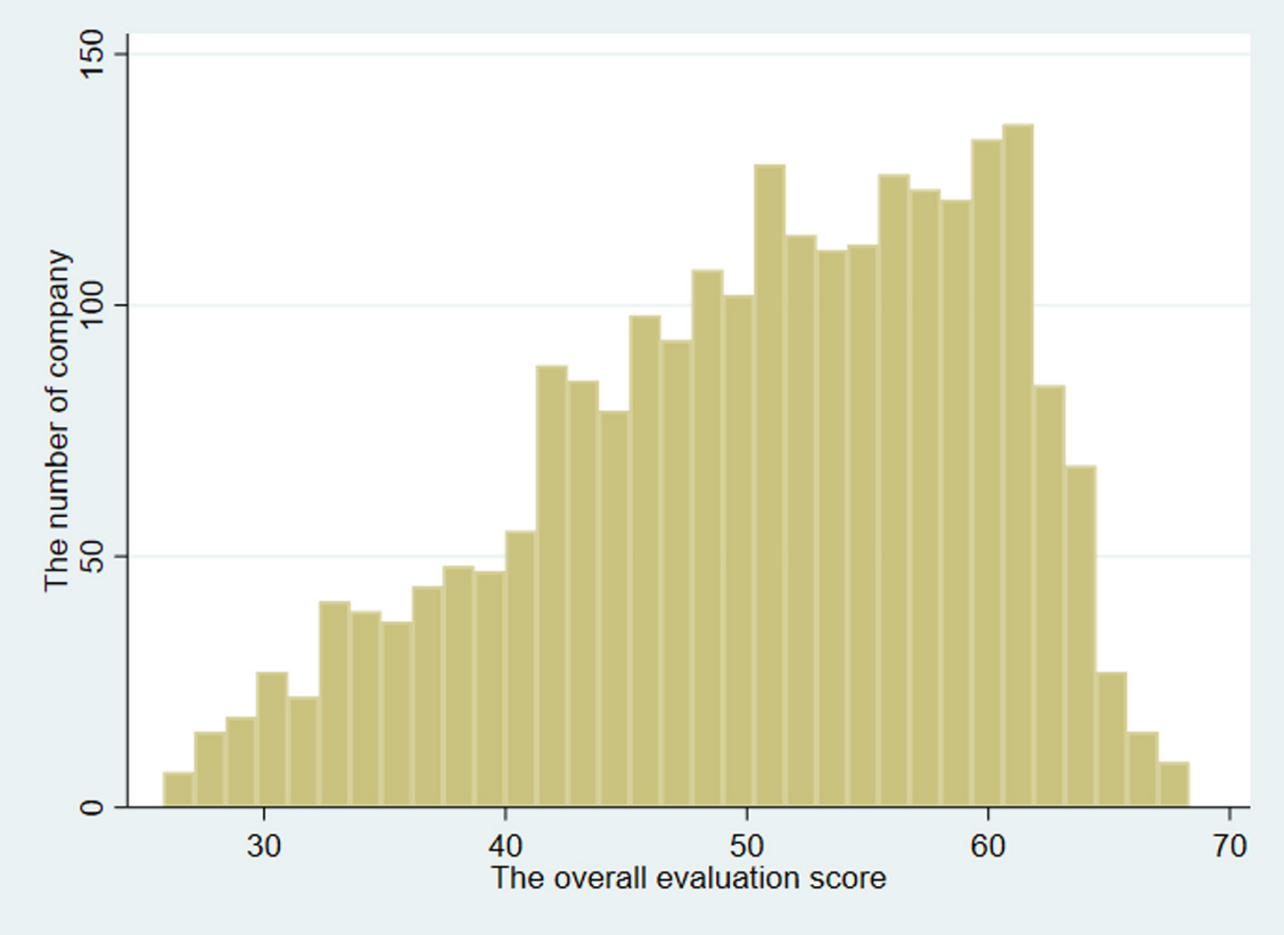
The histogram of the overall evaluation score.

The mean, median, and standard deviation of the overall evaluation score were 50.5, 51.6, and 9.2. The histogram of the overall evaluation score was shown in Figure 1.

The overall evaluation score was divided evenly into three categories, with high ranging from 56.02 to 68.32 points, moderate ranging from 46.95 to 56.01 points, and low ranging from 25.83 to 46.94. There were 784 companies with high scores, 786 companies with moderate scores, and 789 with low scores. Table 1 shows the characteristics of the industries, employee sizes, percentage of those who received annual health check‐ups and stress checks, and operating profit ratio of the target companies in each of these three categories. The most common industry was the manufacturing (*n* = 685), followed by wholesale and retail trade (*n* = 467). In terms of the size of the employee, the largest number of companies had 300–999 employees (*n* = 817).

**TABLE 1:** Characteristics of industry and employee size among the participating companies

	Low score^a^	Moderate score^b^	High score^c^
Total	789	786	784
Industry sector			
Agriculture, forestry, fishing, and mining	0 (0.0%)	0 (0.0%)	4 (0.5%)
Construction	41 (5.2%)	28 (3.6%)	24 (3.1%)
Manufacturing	220 (27.9%)	208 (26.5%)	257 (32.8%)
Electricity, gas, heat supply, and water	3 (0.4%)	7 (0.9%)	13 (1.7%)
Transport and postal activities	32 (4.1%)	21 (2.7%)	28 (3.6%)
Information and communications	84 (10.6%)	108 (13.7%)	107 (13.6%)
Wholesale and retail trade	183 (23.2%)	184 (23.4%)	100 (12.8%)
Finance and insurance	35 (4.4%)	42 (5.3%)	79 (10.1%)
Real estate and goods rental and leasing	20 (2.5%)	20 (2.5%)	17 (2.2%)
Medical, health care, and welfare	42 (5.3%)	36 (4.6%)	26 (3.3%)
Services	104 (13.2%)	110 (14.0%)	102 (13.0%)
Other	25 (3.2%)	22 (2.8%)	27 (3.4%)
Number of employees			
≤99	27 (3.4%)	6 (0.8%)	2 (0.3%)
100–299	198 (25.1%)	146 (18.6%)	66 (8.4%)
300–999	319 (40.4%)	276 (35.1%)	222 (28.3%)
1000–2999	155 (19.6%)	214 (27.2%)	229 (29.2%)
≥3000	90 (11.4%)	144 (18.3%)	265 (33.8%)
Percentage of those who received annual health check‐up			
Median (interquartile range)	100.0 (97.6, 100.0)	100.0 (99.6, 100.0)	100.0 (99.9, 100.0)
Percentage of those who received stress‐check			
Median (interquartile range)	94.7 (88.3, 98.7)	96.0 (90.6, 99.3)	95.7 (92.1, 98.9)
Operating profit ratio			
Median (interquartile range)	4.1 (1.6, 7.9)	4.2 (1.6, 7.7)	5.1 (2.1, 10.2)

^a^ Low score of the overall evaluation score was ranging from 25.83 to 46.94.

^b^ Moderate score of the overall evaluation score was ranging from 46.95 to 56.01 points.

^c^ High score of the overall evaluation score was ranging from 56.02 to 68.32 points.

The median percentage of those who received annual health check‐ups and stress‐check were 100% and around 95%, respectively, in all three evaluation score categories.

Table 2 shows the relationships between the evaluation‐score category and each infection control measurement. The OR of all infection control measurements in the workplace increased as the evaluation score increased.

**TABLE 2:** Relationship between evaluation‐score category and infection control measurement

Overall evaluation score		Infection control measures		Model 1^a^				Model 2^b^			
		n	%	OR	95% CI		*P* value	OR	95% CI		*P* value
	Measures to reduce infection risk among employees obliged to attend the workplace										
		Apply a different shift system than normal to reduce opportunities for contact among employees									
Low score		473	60	ref.				ref.			
Moderate score		607	77	2.04	1.62	2.56	<.001	2.04	1.62	2.56	<.001
High score		711	91	5.10	3.80	6.84	<.001	5.10	3.80	6.84	<.001
		Encouraging off‐peak commuting through staggered work hours and flextime to reduce the infection risk during commuting									
Low score		610	77	ref.				ref.			
Moderate score		674	86	1.53	1.15	2.05	.004	1.53	1.15	2.05	.004
High score		740	94	3.59	2.46	5.26	<.001	3.60	2.46	5.27	<.001
		Distribution of masks required for work and commuting									
Low score		672	85	ref.				ref.			
Moderate score		738	94	2.84	1.97	4.08	<.001	2.84	1.97	4.08	<.001
High score		743	95	3.63	2.43	5.42	<.001	3.63	2.43	5.42	<.001
		Checking and reporting health status (e.g., temperature checks)									
Low score		690	87	ref.				ref.			
Moderate score		753	96	3.16	2.07	4.82	<.001	3.17	2.08	4.83	<.001
High score		769	98	6.08	3.41	10.83	<.001	6.09	3.42	10.85	<.001
		Special considerations for employees with underlying medical conditions that posed a high risk of serious illness in the event of infection									
Low score		317	40	ref.				ref.			
Moderate score		469	60	1.95	1.58	2.40	<.001	1.95	1.58	2.41	<.001
High score		624	80	4.59	3.62	5.81	<.001	4.59	3.62	5.82	<.001
	Equipment factors in the workplace environment										
		Spacing of seats, partitions, and other spatial devices									
Low score		676	86	ref.				ref.			
Moderate score		751	96	3.12	2.09	4.67	<.001	3.12	2.09	4.67	<.001
High score		775	99	9.81	4.87	19.77	<.001	9.81	4.87	19.77	<.001
		Disinfecting areas that many people touch, such as handrails and free‐address desks									
Low score		576	73	ref.				ref.			
Moderate score		721	92	3.88	2.86	5.27	<.001	3.89	2.87	5.27	<.001
High score		762	97	11.65	7.31	18.57	<.001	11.73	7.35	18.72	<.001
		Check and improve ventilation conditions in offices and break rooms									
Low score		636	81	ref.				ref.			
Moderate score		714	91	2.53	1.85	3.45	<.001	2.53	1.85	3.45	<.001
High score		758	97	7.48	4.77	11.72	<.001	7.55	4.81	11.84	<.001
	Other measures										
		Granting of special leave for employees with children in school due to school closures									
Low score		462	59	ref.				ref.			
Moderate score		571	73	1.64	1.31	2.04	<.001	1.64	1.31	2.04	<.001
High score		651	83	2.45	1.91	3.14	<.001	2.46	1.92	3.15	<.001
		Provision of information on preventive measures against infectious diseases									
Low score		673	85	ref.				ref.			
Moderate score		772	98	8.63	4.87	15.29	<.001	8.63	4.87	15.29	<.001
High score		780	99	23.72	8.60	65.38	<.001	23.75	8.61	65.49	<.001

Abbreviations: CI, confidential interval; OR, odds ratio.

^a^ Odds ratio adjusted for industry and company size (categorical variable).

^b^ Odds ratio adjusted for industry, company size and operating profit ratio (continuous variable).

In terms of the measures to reduce the risk of infection among employees who are forced to come to work, the OR of “apply a different shift system than normal to reduce opportunities for contact among employees” among moderate‐score companies was 2.04 (95% CI: 1.62–2.56) and the OR among high‐score ones was 5.10 (95% CI: 3.80–6.84) compared to low‐score ones. The OR of “special considerations for employees with underlying medical conditions that pose a high risk of serious illness in the event of infection” among moderate‐score companies was 1.95 (95% CI: 1.58–2.41) and the OR among high‐score ones was 4.59 (95% CI: 3.62–5.82) compared to low‐score ones.

In terms of the engineering control in the workplace environment, the OR of “spacing of seats, partitions, and other spatial devices” among moderate‐score companies was 3.12 (95% CI: 2.09–4.67) and the OR among high‐score ones was 9.81 (95% CI: 4.87–19.77) compared to low‐score ones. The OR of “check and improve ventilation conditions in offices and break rooms” among moderate‐score companies was 2.53 (95% CI: 1.85–3.45) and the OR among high‐score ones was 7.55 (95% CI: 4.81–11.84) compared to low‐score ones.

## 4 DISCUSSION

The results showed that the higher the overall evaluation score in the HPM Survey, the more measures to reduce the risk of COVID‐19 infection among employees who were forced to come to work and measures to improve the workplace environment were implemented in the corporations participating in the certification program for the Excellent HPM Corporations in Japan. In addition, there was a greater difference in the measures to improve the workplace environment than in the measures to reduce the risk of COVID‐19 infection among employees who are forced to come to work, depending on the overall evaluation score of the HPM Survey.

The level of HPM is based on a comprehensive evaluation using deviation values in four areas: “positioning of OHM in the corporation's philosophy and policies,” “organized frameworks,” “specific systems for implementing HPM,” and “assessment and improvement”.^21^ In order to promote workers' health in the workplace successfully, it has been found that management commitment and leadership support, as well as program enhancement, are important.^16^, ^25^ For this reason, in the certification program for HPM corporations, it is evaluated that the corporations improve programs continuously through a Plan‐Do‐Check‐Act cycle based on the policies of top management. Therefore, the evaluation results of the HPM Survey can be regarded as the status of the corporation's efforts to create a health culture.

Various methods have been used to evaluate health culture,^11^, ^12^, ^13^, ^14^, ^15^ and it has been reported that the presence of health culture is associated with various health‐related outcomes of employees. self‐evaluated performance, altruism, and happiness through health behaviors.^13^, ^16^, ^17^, ^18^, ^19^ As for the effectiveness of health culture in ensuring workers' health in times of crisis, it has been reported that a good health climate, which is closely related to health culture, was linked to job crafting behavior through work engagement under COVID‐19,^26^ and that the presence of health climate in a medical institution with COVID‐19 practices led to a reduction in perceived stress of medical personnel.^27^ However, there have been no reports on the relationship between health culture and the implementation of infection control measures in the workplace during a pandemic, as far as we know. In the present study, there was a clear relationship between corporate efforts to create a health culture and the implementation of infection control measures against COVID‐19. Infection control measures in COVID‐19 protect the lives and health of employees and contribute to business continuity, so it is natural for corporations working to create a health culture to be more proactive in their efforts.

Among the infection control measures against COVID‐19, a difference in the implementation status was observed in the engineering control in the workplace environment, depending more on the overall evaluation score of the HPM Survey than the measures to reduce the risk of COVID‐19 infection among employees who were forced to come. This means that creating a health culture in the workplace had a more positive impact on engineering control in the workplace environment for the prevention of COVID‐19 infection. Among the COVID‐19 measures, it has been reported that the implementation of engineering measures in the U.S. food industry is less than that of administrative measures.^4^ The improvement of the workplace environment, including engineering measures, requires an investment of resources such as human resources and expenses and requires a decision to be made by management. This may be due to the positive influence of health culture in the engineering control in the workplace environment.

It is important to implement infection control measures in the workplace. Spending time in groups can cause clusters,^28^ and companies have been urged by the government to implement telework and other workplace infection control measures. Infection control measures in the workplace also affect individual infection prevention behaviors,^3^ and they are thought to reduce workers' risk of contracting COVID‐19, but to the best of our knowledge, there is not enough information to determine the extent to which these measures have actually reduced the infection rate. In the future, it is necessary to verify the infection rate of excellent companies of health culture.

In the present study, the results of the overall evaluation score based on the responses to the HPM Survey Sheets were regarded as the status of corporate efforts to create a health culture in the corporation. Based on the HPM Survey Sheets, it was found that existing written company‐wide policies and health promotion of workers being on the agenda of management‐level meetings was associated with the participation rate in health promotion programs,^29^ and that the implementation of education for managers was associated with low smoking rates.^30^ However, the relationship between the results of the overall evaluation score of the HPM Survey and the actual results of health promotion has rarely been examined. In the present study, a clear relationship between the results of the overall evaluation score and the implementation status of infection control measures against COVID‐19 was observed, which can be said to be a result that demonstrates the validity of the evaluation method in the HPM Survey.

### 4.1 Limitations

This study is the first to identify the relationship between health climate and infection control measures on a company‐by‐company basis. The strength of the study is that the findings are highly generalizable, using data from approximately 23% of Japan's large companies. Several limitations associated with our study warrant mention. First, since this study mainly focuses on large companies, it is unclear whether a relationship between health climate and infection control measures can be established in small and medium‐sized enterprises. Second, it is possible that the infection prevention measures that companies responded to were only implemented in some of the company's offices. Large companies have multiple workplaces and/or multiple branch offices. Even if the preventive measures are implemented only in some offices in the company, the company answers that they are implemented when the questionnaire asks whether the measures are implemented or not. On the contrary, if a company answered “not implemented” in the questionnaire, it means that they are likely not implementing measures at all or many sites. The low‐score companies answered “not implemented” for all measures more often than high‐score companies. Therefore, it would not have much impact on the results of this study. Third, we conducted this study using the corporate data collected for the purpose of selecting the excellent company of health and productivity management by the Japanese government. Therefore, it was not possible to conduct a comprehensive survey of workplace infection control measures against COVID‐19. Vaccination in the workplace, for example, could not be examined, while other important infection control measures could be discussed. Fourth, the data in this study were the result of responses by company personnel and have not been verified as to accuracy. The survey is designed to select excellent companies in health and productivity management, and applicant companies are encouraged to respond correctly. The rule is that companies keep their responses for 2 years, and if there is any doubt about the answers, additional verification is conducted. In the future, it will be important to verify the validity of the survey by, for example, examining the relationship between the content of the responses to the survey and health outcomes.

## 5 CONCLUSION

The higher the overall evaluation score in the HPM Survey, the more measures to reduce the risk of COVID‐19 infection among employees who were forced to come to work and measures to improve the workplace environment were implemented in the corporations participating in the certification program for the Excellent HPM Corporations in Japan. This study revealed a new finding that the presence of a health culture in the workplace will lead to the appropriate implementation of infection control measures during a pandemic. The company's ordinary commitment to employee health will be effective even in times of health crisis, such as during the outbreak of the pandemic.

## AUTHOR CONTRIBUTIONS

T.N. and K.M. conceived the ideas; T.N. collected the data; T.N. analyzed the data; T.N. and K.M. wrote the first draft; all authors have read and agreed to the published version of the manuscript.

## DISCLOSURE

*Ethics Statement*: The Ministry of Economy, Trade, and Industry obtained consent from all the responding companies to use their data for research purposes. We also signed a written commitment with that ministry to manage the data. We did not handle any individual's personal information. *Informed Consent*: N/A. *Registry and the Registration No. of the Study/Trial*: N/A. *Animal Studies*: N/A. *Conflict of Interest*: The authors declare that there is no conflict of interest.

## Data Availability

The data that support the findings of this study are available from the Ministry of Economy, Trade, and Industry (METI). Restrictions apply to the availability of these data, which were used under license for this study. Data are available at https://www.meti.go.jp/policy/mono_info_service/healthcare/kenko_keiei.html with the permission of METI.
